# A scoping review of men, masculinities, and smoking behavior: The importance of settings

**DOI:** 10.1080/16549716.2019.1589763

**Published:** 2019-04-09

**Authors:** Nurul Kodriati, Lisa Pursell, Elli Nur Hayati

**Affiliations:** a School of Health Sciences, National University of Ireland, Galway, Ireland; b Faculty of Psychology, Post Graduate Program, University of Ahmad Dahlan, Yogyakarta, Indonesia

**Keywords:** Gender and Health Inequality, Masculinities, smoking, scoping review, settings, health promotion

## Abstract

**Background**: In many countries, smoking rates are higher among men than women, highlighting the importance of focusing on factors that influence smoking prevalence among men. Expressed masculinities occur within settings that can influence men’s perspectives and behaviours towards smoking.

**Objectives**: To provide an overview of key aspects of how masculinities underpin men’s behaviours regarding tobacco smoking.

**Methods**: The Health, Illness, Men and Masculinities framework was used to develop a synthesis of masculinities captured in published articles about men’s smoking behaviours. Five databases (PubMed, Medline Ovid, Embase, CINAHL and PsychINFO Ovid) and Google Scholar (up to April 2016) were searched using keywords derived from three concepts: men, smoking and health. In total, 351 articles that focused on smoking and used/implied masculinity concepts were identified. These underwent a two-stage screening process applying inclusion/exclusion criteria, first titles/abstracts and then full-text. Data from 45 selected articles were extracted and charted.

**Results**: Regions with high prevalence of smoking among men, such as South East Asia and the Western Pacific, had a disproportionate number of studies on masculinity and smoking, with less exploration of masculinity as a protective factor, especially for young people, and men-specific settings to support non-smoking behaviour.

**Conclusions**: Incorporating masculinity in future settings-based approaches to smoking-related health promotion programmes has the potential to reduce smoking prevalence among men.

## Background

Smoking remains a major public health issue worldwide [1], with a dramatic increase in numbers from 721 million in 1980 to 967 million in 2012 [2]. This high prevalence has led to vast numbers of health-related outcomes; for example, 143.5 million Disability Adjusted Life Years in 2013 [3] and 12% of total adult mortalities were attributed to smoking [4].

Ng et al. [2] found that despite the general trend of smoking prevalence being higher among men than women, the ratio of men–women smokers varied across regions and demographic groups. In some countries (e.g. Korea, China and Indonesia) the difference is marked, with smoking among men outnumbering that among women by more than 10:1. This difference remains after stratification by age group, and boys were significantly more likely than girls to smoke cigarettes in many countries [5].

Despite the increased prevalence of smoking, positive changes have been noted. Reductions in prevalence among men have been reported in high-income countries/regions such as Canada, Hong Kong (China), Japan and Singapore [1]. Several trials have shown that smoking cessation rates tend to be higher for men than for women [6,7]. However, little evidence is available on whether such findings reflect circumstances of the respective study populations. For example, Luo and Xie [8] found that the reduction in smoking prevalence among men reported in China represented a decrease in the initiation of smoking among men, rather than smoking cessation. Furthermore, underlying reasons for reported smoking cessation or reductions in initiation remain unclear.

A current focus of health promotion interventions for tobacco use involves modifying the external environment, such as increasing tobacco taxes, creating smoke-free environments, package warnings and advertising bans, as suggested by the World Health Organization (WHO) [4]. However, such tobacco control policies have been implemented among less than 40% of the world’s population [9], leaving a large number of people unreached and unprotected. In areas where such regulations have been implemented, tobacco taxation and pricing are considered an effective regulation technique for short-term effects, but evidence is lacking regarding any long-term impact in reducing prevalence, especially among men [10].

Diclemente et al. argued that successes from the structural controls above might be complemented by an application of behavioural theories [11]. These authors argued that sustained change would only be possible if there are sufficient internal and external influences. While the extension of the policy to the 60% of the world population currently unprotected by policy-level interventions is ideal, there remain higher relative rates of smoking among men in countries where these controls exist. Therefore, interventions should also consider men’s internal dynamics to complement current smoking policies.

In this review, the internal dynamic focuses on masculinities, or the values associated with being men. This focus was chosen because, despite the majority of smokers being men, little is known about how ‘being a man’, as a social construct, relates to smoking behaviour. In much of the literature, capturing gender as a factor in smoking behaviour involves the use of a simple male-/female-based assessment, particularly in cross-sectional studies, resulting in such data tending to reflect biological differences rather than gender’s complex social constructs. In terms of masculinity, a growing body of literature on the nature of masculinities generally accepts the term as representing multiple forms. In many cultures there is a well-accepted masculine ideal, which is passed from one generation to another, even though many men are not able to meet its standard entirely. David and Brannon called this ideal ‘culture’s blueprint of manhood’ [12]. Connell’s [13] definition of masculinities concerns the position of men in a gender hierarchy of multiple entities. Interactions among these entities are complex and can be contradictory. Connell argues that the majority of studies refer to hegemonic masculinities, which she characterised as a dominant type of masculinity in comparison to other subordinated or marginalised types. These other types are categorised by practices of masculinities that vary as they interplay with other factors such as race, class, sexual orientation and region, including, for example, men who are gay or have lower social status. Properties and practices associated with the hegemonic type have been framed as traditional masculine characteristics. These have been summarised by Courtenay [14] as being more prone to risk-taking and unhealthy behaviours, as well as being less willing to seek support when needing help. However, even though a large number of men do not follow the hegemonic pattern, most still benefit from and are complicit with the established gender hierarchy [13].

Because of this hierarchy, Kimmel [15] argued that within cultures, men are invisible. This, he argued, is because men are predominantly regarded as maintaining a more powerful position in most societies, and as such their particular experiences are considered the norm and therefore studied less frequently. However, men have health problems specifically related to risk-taking behaviours, such as smoking, violence and alcohol [16]. Despite this, men’s health as a gendered issue has only gained attention very recently. A recent WHO European region report [16] acknowledged men’s specific health problems resulting from the social construction of their gender. On the basis of this, guiding principles and priorities for actions to improve men’s health were developed in the report as a means to contribute to gender equality.

The characteristics of multiple masculinities share with health promotion’s main framework for action – the Ottawa Charter [17] – the importance of settings, thus providing a clear framework for the focus of action for smoking interventions for men in places where they tend to spend their time, for example, at their workplace, with their family or other traditional specific settings among defined populations, such as the military. In this latter setting, smoking prevalence has been found to be relatively high compared to the general population [18], indicating the importance of studying the impact of masculine characteristics on men’s smoking behaviour.

In addition to particular settings, when considering men’s health behaviours, the Health, Illness, Men and Masculinities (HIMM) framework [19] offers a guide to understanding how multiple masculinities intersect other social determinants of health throughout the course of life. Men experience a wide range of social diversity that influences their understanding of what constitutes being a man. The HIMM frames how their practised masculinities also vary in response to age, race, cultural and occupational status across male lifespans. These multiple interacting factors may affect smoking behaviours among men in different ways. In this report, it is argued that the articulation of the complexity of masculinities and their relation to smoking has the potential to provide more focused approaches to developing policy and other interventions targeted towards men.

This review aims to provide an overview of key aspects of how masculinities underpin men’s behaviours regarding tobacco smoking. Specific objectives were to conduct a systematic search of studies on smoking behaviour that incorporated or implied theories of masculinities; to map out the general characteristics of included studies with respect to quality, methodological approach, location, setting and age group; and to identify relevant key themes that capture relationships between men’s smoking behaviours and masculinities. A further objective was to identify gaps in the knowledge and to propose recommendations based on a deeper understanding of masculinities in relation to men’s smoking behaviours.

## Methods

A narrative approach was taken in scoping the literature [20,21]. The scoping process followed the methodological framework proposed by Arksey and O’Malley [22], and incorporated a thematic synthesis of findings from included articles.

### Search strategy

A systematic search was conducted across five databases: PubMed, Medline Ovid, Embase, CINAHL and PsychINFO Ovid. Additional searches were performed in Google Scholar and the reference lists of previously identified articles to find more recent studies (April 2016). No limitations were applied at this stage, to ensure wide inclusion of available publications. The search strategy was developed by the main author, in consultation with a librarian.

Three key concepts, ‘men’, ‘smoking’ and ‘health’, were used to develop keywords, and the process was developed in PubMed, where suitable Medical Subject Headings (MeSH) terms were identified. Resultant keywords were used to retrieve relevant articles (Table 1).

**Table 1.** Keywords employed in each database during the literature search.

No.	Keywords	No.	Keywords
1	Men	9	‘Health literacy’
2	Man	10	‘Health education’
3	Masculinity	11	‘Health knowledge, attitudes, practice’
4	Smoking	12	1–3/OR
5	‘Tobacco use’	13	4–6/OR
6	‘Smoking cessation’	14	7–11/OR
7	‘Health behaviour’	15	12 AND 13 AND 14
8	‘Health promotion’		

*Source*: Databases (PubMed, Medline Ovid, Embase, CINAHL and PsychINFO Ovid) and Google Scholar.

Building sets of keywords and checking search results were conducted iteratively, until the articles resulting from the searches were considered relevant and the numbers reasonable.

### Screening process

Inclusion and exclusion criteria were applied to titles and abstracts, followed by full-text screening. These steps were initially conducted by the main author using EPPI-Reviewer 4 (V.4.5.1.0, University College London). The second and third authors were consulted in cases of uncertainty about inclusion. Abstracts were considered for inclusion if their main study subject concerned smoking behaviour among men and/or theories of masculinities. In applying criteria, masculinity was defined as a social construct experienced and expressed at a specific time, culture and locale that has a significant influence on determining how men behave [13]. Articles were screened for whether they contained perspectives on masculinity values in the analysis, availability of full-text, the empirical nature of the study and accessibility in English. Selection was not limited by the type of tobacco. Exclusion criteria were irrelevant topics, unavailable abstracts or full-texts and articles in languages other than English. Studies with smoking as a predictor of certain diseases or studies about men that were not specifically concerned with their smoking behaviours were excluded.

### Data extraction and analysis

**Figure 1.**
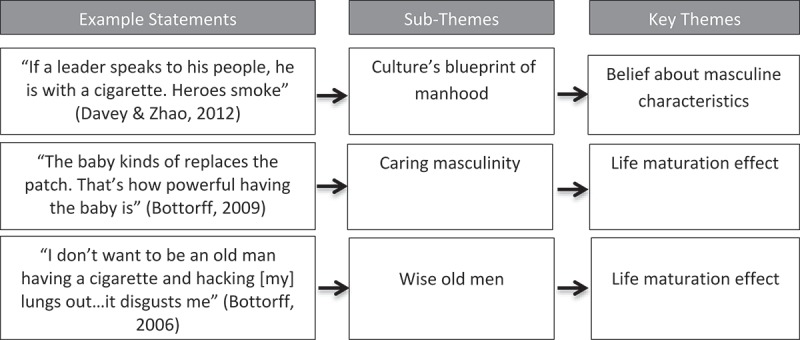
Example of the emergence of themes and subthemes from related statements.

Data extraction and charting were conducted to start the synthesis and data analysis. The charting extracted basic data, including year published, research question(s), sample size, response rate (for quantitative studies), study population, respondents’ age, settings and the main results. The next step synthesised findings into meaningful thematic content. Initial development of themes used MS Excel to paste relevant text from articles into categories based on similarity of theme. This process was underpinned by identifying life events, settings and interventions specific to men, and aspects of their expressed masculinities related to smoking behaviour that intersected with other health determinants, such as age, race, geography and education, as described by the HIMM framework. The categories were coded, and their contents reviewed as an iterative process. Latent terms were posed for the groupings, taking account of the structure of the HIMM framework. Notes were taken where categories intersected or diverged. This process was primarily undertaken by NK. Figure 1 presents examples of statements from reviewed articles that are illustrative of the sub and main themes. Reviewing of categories and rereading of manuscripts formed an iterative process conducted by NK with consultation throughout where any ambiguities arose with LP and ENH. No other software was employed in this process.

Quality of included qualitative and quantitative articles was assessed using the critical appraisal instrument developed by Hawker et al. [23]. The instrument comprises nine quality criteria (abstract and title, introduction and aims, method and data, sampling, data analysis, ethics and bias, results, transferability/generalisability and implications and usefulness). Each criterion has four score options (0–3) corresponding to very poor, poor, fair and good. Total scores vary from 0 to 27. In this review, these scores were classified into three quality groups: low (<10), medium (10–18) and high (>18).

## Results

**Figure 2.**
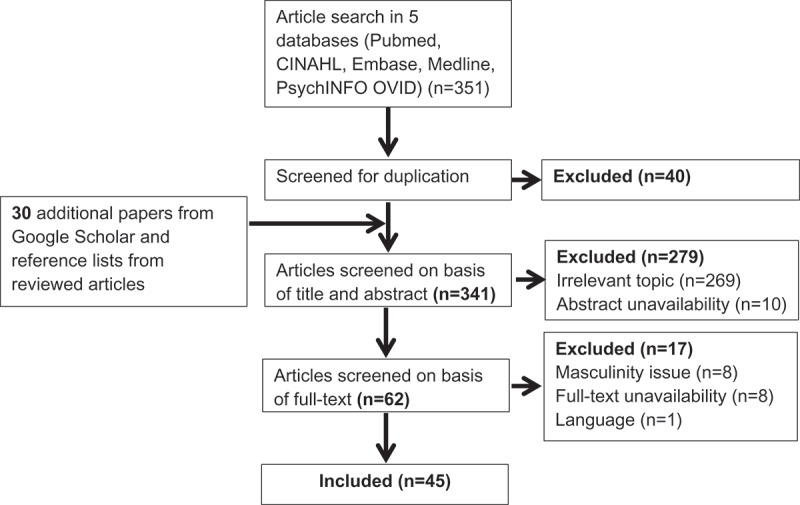
Flow diagram of articles identified and selected.

Figure 2 shows the outcomes of the search process. In total, 351 articles were retrieved; 40 were excluded because of duplication. An additional 30 articles were identified by searching Google Scholar and the reference lists of previously identified articles.

Selection based on the title and abstract screening excluded a further 279 articles, resulting in 62 articles eligible for full-text screening. Following full-text screening, 17 articles were excluded, leaving 45 articles for inclusion in the review. The results of the quality assessment showed that two articles out of 45 were of medium quality, and all others were of high quality.

### General characteristics

A summary of the general characteristics of the included studies is presented in Table 2. Most studies were conducted in the region of the Americas (53.3%), with only one each in Africa and South East Asia. There was an increasing trend of research focusing on masculinity and smoking behaviour over the last 30 years, peaking during 2007–2016 (62.2%). The earliest of the included studies was conducted in 1990 in Kenya, Africa. All studies focused almost exclusively on smoking tobacco, with only one smokeless tobacco study included.

**Table 2.** Summary of characteristics of included studies.

No.	Author (year)	Region	Age group	Settings	Key themes
1	Cronan, Conway (1991)	The Americas	Adult	Education	BMC, EES, PA
2	Everett, Gage (2005)	The Americas	Adult	Community	LME
3	Everett, Bullock (2007)	The Americas	Adult	Community	LME
4	Maxwell, Garcia (2007)	The Americas	Adult	Community	EES
5	Kim (2008)	The Americas	Adult	Community	EDS
6	Badr, Moody (2005)	Eastern Mediterranean	Adult	Workplace	BMC
7	Nazary, Ahmadi (2010)	Eastern Mediterranean	Adult	Education	EES
8	Gao, Zheng (2011)	Western Pacific	Adult	Workplace	EDS
9	Lin, Sloan (2015)	Western Pacific	Adult	Community	LME
10	Loke, Mak (2012)	Western Pacific	Adult	Health services	LME
11	Stanton (2004)	Western Pacific	Adult	Health services	LME
12	Blackburn, Bonas (2005)	Europe	Adult	Community	LME
13	Schei, Sogaard (1994)	Europe	Adult	Workplace	BMC
14	Kaplan, Carriker (1990)	Africa	Adult	Community	EES, LME
15	Greaves, Oliffe (2010)	The Americas	Adult	Community	EES, LME
16	Bottorff, Oliffe (2006)	The Americas	Adult	Community	BMC, LME, EES, PA
17	Bottorff, Radsma (2009)	The Americas	Adult	Health services	BMC, PA, LME
18	Bottorff, Oliffe (2010)	The Americas	Adult	Health services	EDS, BMC, PA, EES
19	DeSantis (2002)	The Americas	Adult	Community	BMC, PA
20	Dutta, Boyd (2007)	The Americas	Adult	Community	BMC
21	Johnson, Oliffe (2009)	The Americas	Adult	Health services	BMC, EES
22	Kim, Son (2005)	The Americas	Adult	Health services	BMC, EES, EDS
23	Kim, Nam (2005)	The Americas	Adult	Community	EDS, EES, LME
24	Kwon, Oliffe (2014)	The Americas	Adult	Health services	BMC, LME, PA
25	Kwon, Oliffe (2014)	The Americas	Adult	Health services	BMC, EDS, LME
26	Oliffe, Bottorff (2008)	The Americas	Adult	Health services	BMC, PA
27	Oliffe, Bottorff (2010)	The Americas	Adult	Health services	BMC, EES, PA
28	Oliffe, Bottorff (2012)	The Americas	Adult	Community	BMC, PA, LME
29	Kim, Son (2005)	The Americas	Adult	Community	EES, LME, PA
30	Tu, Walsh (2000)	The Americas	Adult	Community	BMC
31	Ng, Weinehall (2007)	South East Asia	Adolescent	Community	BMC, EES, LME, EDS, PA
32	Davey, Zhao (2012)	Western Pacific	Adolescent	Education	EES, PA, BMC,
33	Wakefield, Reid (1998)	Western Pacific	Adult	Health services	LME, BMC
34	O’Brien, Hunt (2009)	Europe	Adult	Community	BMC, LME
35	Cortese, Ling (2011)	The Americas	Adult	Community	BMC
36	Cable, Meland (1999)	Europe	Adult	Community	PA, EES, LME
37	Ziebland, Fuller (2001)	Europe	Adult	Health services	LME
38	Schwappach (2009)	Europe	Adult	Community	EES
39	Valera, Cook (2014)	The Americas	Adult	Community	EDS
40	White, Oliffe (2012)	The Americas	Adult	Community	LME
41	Bottorff, Haines-Saah (2012)	The Americas	Undefined	Community	BMC, EES, EDS,
42	Morrow, Barraclough (2010)	Western Pacific	Undefined	Community	BMC, LME
43	Okoli, Torchalla (2011)	The Americas	Undefined	Health services	NA
44	Bottorff, Haines-Saah (2014)	The Americas	Undefined	Community	BMC, LME, EES
45	Roberts (2006)	Europe	Undefined	Community	BMC

Themes BMC: Masculine Branding; EDS: External Discouragement to smoke; EES: External Encouragement to Smoke; PA: Psychological Attachment; LME: The Life Maturation Effect. Regions were based on UN categorisation.

A majority of the included studies were qualitative, providing different levels of findings, including ethnographic data derived from exploration of photographs and magazine articles. The targeted populations were evenly spread across age groups, although only five out of 45 studies focused on boys (teenagers). One study with women respondents was included, because it aimed to understand men’s smoking behaviour from the perspective of women. The included quantitative studies focused on smoking among men, with varying emphasis on smoking behaviours. Some focused on special life events for men; for example, the transition towards becoming a father. Two were specifically designed to collect information in places characterised as having strong masculinity values (e.g. military camps). All included quantitative studies recruited respondents aged 18 years and over.

The study populations provided rich accounts of how masculinities varied across the life course and their differing relationship to men’s smoking behaviours. A large proportion of these accounts were dedicated to understanding the adult compared to the younger population of men. Most were conducted in community settings (64.4%). The lowest percentage comprised studies conducted in educational settings (school or training), followed by health services (28.9%).

### Key themes

Following the contextualisation of this review within the theoretical HIMM framework, the key themes identified, representing how different aspects of masculinities relate to men’s smoking behaviours, were *belief about masculine characteristics* (BMC), *external discouragement to smoke* (EDS), *external encouragement to smoke* (EES), *psychological attachment* (PA) and *the life maturation effect* (LME).

The first key theme, BMC, forms a starting point to understand smoking behaviour in the context of the HIMM framework, as it underpins much of the relationship between men’s external and internal influencers regarding smoking. David and Brannon’s [12] phrase ‘culture’s blueprint of manhood’ is used within this theme, to represent how this blueprint has been captured and strengthened in popular media [24], then used to reinforce acceptance of smoking among men [25,26]. Reciprocally, some men perceived smoking as a signifier of their masculinity [27–29] by not displaying ‘feminine and weak’ attributes where there are cultural prohibitions on women smoking [28] and by young men perceiving it as a route to their adulthood [30]. Gay men also perceived smoking as part of their identity, feeling it made them appear more attractive [31].

The BMC theme underpins the way men respond to their social environment and the stressors within it, as captured by the themes EDS, EES and PA, and therefore interacts with them. BMC itself is a dynamic that changes along men’s course of life, as reflected in its interaction with the theme LME.

The second and third key themes, identified as EDS and EES, reflect how external environments have shaped and influenced men’s decisions both positively and negatively on whether to smoke or not. These capture situations where men perceived the need to smoke in order to be accepted by their peers, or conversely to avoid smoking where it does not fit within their family’s or society’s value systems. However, the external environment was mainly portrayed as a factor that encourages men to smoke. In such circumstances, men tend to conform to maintain social harmony [26,28,32], and enable engagement in certain groups [32], for example, among students [33,34], military men [35] or gay men [31].

Some men depended on smoking to start or maintain interactions with others, and this influenced their smoking behaviour in three ways. First, in regions where smoking prevalence is high (e.g. China and Indonesia), it forms an important part of men’s social interactions [26,28]. Second, some men maintained the influence of their previous social interactions after migrating to a different country by smoking, to keep a sense of their cultural identity [36]. Third, younger men or those with lower social status emulated prevalent smoking behaviours among adults or among people with higher social status, to help them to feel more socially acceptable [30]. The settings in which men experienced masculinities that influenced their smoking behaviour in both themes involved interactions with family and within cultural groups, where it was used as a means of maintaining inclusion.

The fourth theme, identified as PA, captures internal drivers for smoking among men. It denotes the influence of the internalisation of more harmful aspects of masculinity in relation to their smoking. The theme also captures how, throughout their course of life, men may rationalise their smoking behaviour in complex ways that involve self-narratives about their attachment to smoking, to cope with stressful life circumstances [28,37,38]. School-aged boys perceived smoking as a means of coping with school-related stress, feeling it calmed them [26,28]. During adulthood, smoking was thought to be necessary to maintain the emotional stability required for being a man and to cope with work-related stress [29,35,39], and during difficult periods of adapting to a new role as a father [27]. Additionally, men’s partners may encourage smoking to acknowledge or reward them for being a good father [40] and as a means of reducing any negative behaviours that arise when their partners attempt to quit [27]. The settings from which this theme was derived involved adult men and boys engaging with perceived stressful demands in various places such as schools, workplaces and the home, which shows the relevance of discussing stress within settings in relation to smoking among men.

Lastly, the LME represents the dynamic nature of masculinity, often expressed as needing a more moderate smoking behaviour as men age. This theme captures the phenomena of young men holding a belief that they have physical resilience to the harmful effect of smoking [27,28,32], then starting to re-evaluate their smoking-related behaviours when they enter fatherhood and living modestly in keeping with their age as they get older [38]. When smoking is perceived as such an integral part of being a man, even a new role that is considered to be incompatible with smoking, such as being a father, may not necessarily lead men to quit smoking [27]. The settings for this theme were inevitably dependent on the stage of life, with the school setting being important in boys’ interactions with their peers, and the family, home and workplace being of importance later in life.

## Discussion

This review adopted a narrative approach to scoping the literature, enabling examination of a topic that is heterogeneous in nature that has not previously been extensively reviewed [41]. It aimed to identify key aspects of masculinities that underpin men’s tobacco smoking behaviours. Arksey and O’Malley's [22] systematic approach to scoping literature is a widely used guide [42,43] for such reviews, and its use enabled mapping of the characteristics and quality of relevant published studies and identification of significant gaps in the literature. Unlike systematic reviews, assessment of quality is debatable in a scoping review and is, therefore, less frequently performed [41]. Its use in the current review illustrated the good quality of the literature featured. Incorporation of a thematic synthesis enabled the identification of key themes relating aspects of masculinities and smoking behaviours among men.

### General characteristics of the studies

Mapping the characteristics of the studies shows that the inclusion of masculinities within smoking studies is a relatively new approach that has only gained momentum as a distinct research activity in recent years. Given how recent this focus has been, it is not surprising that it has been limited to certain regions, age groups and settings. Included articles tended to focus mostly in the north-western region, particularly the Americas. This region is referred to as metropole by Connell [44] and described as ‘former imperial powers with continuing postcolonial connections, and the centres of military, communication and intelligence networks’, remaining the main source of current theories and knowledge bases. In order to assess how smoking behaviours and the diverse adoption, rejection or adaptation of historically promoted masculinities and their more recent influence by neoliberal globalisation interact as social determinants of health in countries of the south-eastern region, as opposed to the north-western, a greater focus on their current sociocultural perspectives is necessary. This is particularly important, because approaches to interventions still predominantly derive from the north-western region.

### Key themes

Most studies reviewed associated smoking with the traditional masculine characteristics as defined by Courtenay [14]. These characteristics often describe hegemonic masculinity, a type of masculinity that legitimates men’s dominant position relative not only to women but also to other subordinated masculinities [13]. In this context, men reported in those articles often associated their smoking behaviour with characteristics such as being powerful [26], being emotionally stable [27], being in control [29] and having self-reliance [29]. This review suggested that these masculine-related characteristics and men’s beliefs about them are fundamental aspects in understanding why smoking prevalence is much higher in certain age groups and how men use smoking to cope with stress and deal with their social environment, as described in the themes of life maturation effect, psychological attachment and external encouragement/discouragement to smoke.

In light of Kimmel et al.’s [45] argument regarding the invisibility of men in studies in general, it is not surprising that this review identified a paucity of smoking studies related to men and masculinities. This persisted, despite smoking prevalence having been much higher among men in many countries for decades [46]. In this context, the qualitative studies reviewed contribute important perspectives on how men relate their smoking behaviour with their masculinities. Of note is that some men define their masculinity by being different from women and attach to a traditional masculinised ideal of smoking to support their expression of masculinity [26,28].

Among the few studies on smoking and masculinity, aspects of the hegemonic type have been their main focus, even though few men can fulfil this standard, putting them in subordinated or marginalised positions relative to its idealised characteristics [13]. From the studies reviewed, it is unclear how these other forms of masculinities affect men’s smoking behaviour, as only one article focusing on gay men [31] represented a subordinated masculinity. However, the findings of that study were similar to those associated with hegemonic masculinity in that its respondents perceived smoking as part of the male gay scene [31].

Despite men being studied only rarely as a subgroup of the population [15], their smoking problem needs to be addressed to the same extent as women, particularly in relation to smoking. This is especially so in the context that while smoking among men is more than five times higher than among women [2], men are not always the direct smokers. In fact, the prevalence of experiencing second-hand smoke derived from 192 countries is 34% and 33% for women and men, respectively [47]. Thus, the total burden of second-hand plus first-hand smoking for men is extremely high. While there has been a change in patterns for both men and women [48], failure to consider the relative prevalence of smoking between these groups will result in any attempt to differentiate gendered smoking patterns being misleading, and diverts from seeing the main burden of smoking, which is more prominent among men rather than women.

It is unclear whether the practice of second-hand smoking among men could be considered complicit masculinity (they do not smoke but do not stop their colleagues from smoking) or subordinate masculinity (they do not meet the standard of hegemonic masculinity). Alternatively, second-hand smoking might be framed as an expression of hegemonic masculinity, in a positive way, by representing those who have willpower, commitment and authority to decide not to smoke and not to be influenced by their environment, as an interpretation of hegemonic masculinity does not always indicate hierarchy [49]. Such research would be interesting to conduct in settings representing the traditional values of hegemonic masculinity, such as the military, where exploration of smokers and non-smokers in relation to aspects of their masculinities could be a valuable contribution to the knowledge of this area, particularly for framing and presenting health messages for men related to smoking behaviours.

In terms of incorporating aspects of masculinities into programmes within different cultural settings, some programmes have been established to engage with men to prevent domestic violence. Rutgers’ Mencare+ programme [50] and Promundo’s International Men and Gender Equality Survey (IMAGES) [51] are illustrative approaches to incorporating an understanding of men’s attitudes, areas of resistance and mapping of continuing challenges of engaging men and boys in order to advance gender equality. A similar approach could be adopted for smoking cessation programmes.

Given that men’s unique circumstances influence what they are, a deeper understanding of the social construction underpinning gendered differences requires a translation of how constructs of masculinities contextually produce different perceptions, identities and behaviours towards smoking between men and women. For population-based surveys this requires well-designed methodology incorporating questions capturing masculine characteristics, as reflected by the key themes in this study. Thus, a well-conceptualised masculinity study on smoking might also consider conducting such surveys in a specific setting representing hegemonic masculinity, such as military camps. A deeper understanding of masculinities will also consider critical stages of men’s life, such as fatherhood, to complement smoking studies, focusing on different ages or how certain occupations might impact fathers’ involvement with their children and development of their father identity, to protect their children from the harm of second-hand smoking [52].

A survey of men that was conducted across some countries in the Asian Pacific region incorporating aspects of masculinity characteristics was conducted as part of UN Multi-Country Study on Men and Violence. However, only the Indonesian sample included questions on smoking [53]. To enhance research into quantitative aspects of smoking and masculinities, an extension of such surveys with the inclusion of smoking-related questions in other countries would be beneficial in providing health authorities with data relevant to targeting cessation programmes among men. Aspects of the key themes identified in this review need to be incorporated into surveys to explore the link between masculine characteristics and smoking prevalence, particularly in cultural groups where these are very high among men.

The HIMM’s framing of masculinities interacting with other health determinants underpins the themes of EES and EDS, in relation to men’s external environments, and the theme of PA in relation to their internal drivers. Important findings related to factors that were instrumental in men’s smoking behaviours were captured in these themes. Internal self-narratives formed by men’s beliefs about masculine characteristics were instrumental in men’s decisions to smoke. These narratives impact men’s coping and interactional skills within their external environments, resulting in a lack of healthy coping strategies and low social skills that instrumentally mediate men’s smoking behaviours. This was illustrated by Kim et al.’s study [30] where men depended on smoking to initiate or maintain interactions with others. This dependence also indirectly reflects their capacity to reject smoking in their social settings.

Similarly, Andretta et al. [55] reported low social skills among drug users, with men’s social interaction skills significantly lower in comparison to women. This highlights the need for studies on smoking among men to include aspects of social skills, to determine which operate to encourage or discourage smoking within different cultural settings, as a preliminary round to develop more nuanced interventions incorporating the enhancement of men’s social skills. Environments could influence men’s smoking behaviour positively. In this study, parents play a role in at least delaying boys from smoking, and spouses often have different strategies to regulate men’s smoking behaviour. Practising certain religions could also beneficially help men to consider their smoking behaviour; for example, Korean men who were practising Christians smoked less than those who were not [54]. The use of tobacco has been debatable among Muslims, as scholastic rules stipulate that Muslims are discouraged (*mukrooh*) or prohibited (*haram*) from smoking. However, there is no halal (permissible) certification in any cigarette. Therefore, it is also important to bring the discussion and research into religious settings to understand how to optimise men’s beliefs to motivate them to stop smoking.

That some men perceived smoking as a part of their coping strategies for stress reduction [55] supports their traditional beliefs about masculine characteristics of not requesting help from others, keeping their emotions stable, while expanding and maintaining their social networks. Both smoking initiation and maintenance were also perceived as a way of alleviating stress among individuals during men’s early adulthood [26,28], indicating unhealthy coping strategies. This highlights that interventions directed towards smoking cessation or prevention should not overlook these instrumental factors. Studies, therefore, need to explore alternative positive coping strategies among men smokers. A study by Bindu et al. [56] had started this exploration among men smokers in India, and reported that among those who perceived their stress to be high, problem-solving, positive distraction, acceptance and faith were used as protective coping strategies. Larger-scale and more robust studies within regions of high smoking prevalence among men need to be conducted to determine the extent and value of such coping strategies within different cultural settings.

Several articles included phenomena that depicted men’s beliefs about their masculinities at different developmental stages in the course of life [26,28,38,57]. These were captured in the theme of LME, reflecting how behaviours and perceptions regarding smoking changed with age. The belief of young men to have physical resilience to the harmful effect of smoking was supported by a systematic review study of smoking initiation among Asian adolescents. Boys (ages 10–14) were more likely to have initiated smoking, and the majority had their first experience in junior high [58]. Smoking studies or interventions incorporating masculinity will require consideration of different stages of men’s life course. This knowledge could be translated into age-appropriate masculinity-scripted messages regarding smoking. For example, during adolescence, the messages could focus on physical fitness [59], and during men's productive ages on being a good provider. For older men, a focus on independence and autonomy, to avoid dependence on others, may be more appropriate [60].

Given that many men initiate their smoking behaviour while young [61], it is important to develop interventions that are appropriate to that age. Studies or interventions focusing on boys might develop their programmes within school settings with strong contextualisation of their indestructible young age values. Boys like to develop their body strength, and therefore physical activity might be an important aspect to be employed, as it serves as a protective factor against smoking [59].

### Gaps and recommendations

The systematic search employed in this review made it possible to identify significant gaps in the literature. Four significant gaps were identified. First, as this review did not limit its search by geographical area, we could identify that regions with a high gender prevalence ratio (men–women) outside of the Americas were very under-represented. Second, there was little exploration of masculinities as protective factors against smoking for men; those that did were limited to focusing on the impacts of men becoming fathers. Third, very few studies incorporated instrumental aspects of smoking behaviour among men, such as smoking, as a stress-reducing strategy or means of social interaction. Fourth, there was a lack of literature on smoking interventions focusing specifically on men.

In terms of geographical area, very few studies focused on countries of the south-eastern region as described above or the consequences of constructed masculinities in that region to smoking behaviours among men. Of particular note, regions with a high prevalence of men smoking, such as South East Asia and the Western Pacific, had produced few articles on masculinity and smoking. Despite a male–female smoking ratio in South East Asia of 16:1 [46], only one article identified in this review concerned this region. This gap underpins the first recommendation, which calls for more studies on masculinity and smoking in countries where smoking among men is much more prevalent than among females.

Second, a focus on aspects of masculinities as a protective factor against smoking is required. A main protective factor highlighted in the reviewed studies was fatherhood. However, the exploration of other potential aspects of masculinities that might serve as protective factors against smoking among men is necessary.

Third, the included studies indicated some men perceive the need to smoke in order to engage in social interactions, and to cope with stressors. This highlights the third recommendation that smoking studies and interventions need to incorporate aspects of healthy coping mechanisms and social skills as integral parts of smoking control and interventions among men.

Fourth, interventions need to be designed specifically for men, as they have the potential to accelerate smoking cessation among men, due to their responsiveness to those smoking interventions [6,7]. Assisted ‘quit smoking’ programmes and counselling need to be adjusted with regards to masculinity values, as some men will prefer quitting unassisted and lay knowledge gained directly from personal experience and rely on their own motivation, willpower and commitment, which reflects their self-identity [62,63].

### Strengths and limitations of this scoping review

The inclusion of qualitative articles as the main type of study in this review provided an in-depth view to gain a better understanding of interconnections between masculinities and smoking behaviours. The review limits its relevance to wider settings, due to the inclusion of English-only articles. An ideal situation would be to include studies employing languages other than English. However, we limited our study to English-based manuscripts, as it is the main language used for scientific publication. On the basis that it potentially provides many studies from across the world; various cultures represented in the studies reviewed were originally from a range of different countries, so to some extent this was achieved. The local cultures represented in the reviewed studies were from several different cultural groups that had not necessarily originated in the study location. The extent to which these represent their cultural origins could not be discerned; however, there was a surprisingly similar adoption of smoking as a representation of hegemonic masculinity, regardless of whether the cultural group had moved to other locations or were in their country of origin. The review particularly under-represents studies from south-eastern regions, and this was identified as a significant gap in the literature in a region where the highest prevalence of smoking occurs among men.

## Conclusion

Despite having an extremely high prevalence of first-hand and second-hand smoking, studies and interventions targeting men are considered to be very limited. This is evident in that men, indeed, are invisible, as argued by Kimmel [15]. The most significant contribution of this review is to understand the underlying reason for smoking problems among men, by employing the multiple concepts of masculinity theories. This review, therefore, provides an initial step in drawing the attention of the international scientific community to the relationship between smoking and masculinities.

It is clear that smoking is a signifier and an influencer of the dynamics of masculinity throughout men’s course of life. As such, their decisions on smoking are influenced by their understandings of what constitutes appropriate masculine behaviour. The dynamics of this relationship operate within particular settings. This highlights the importance of supportive settings in enhancing and promoting aspects of masculinities that are protective against smoking throughout men’s course of life.

Regions with a high prevalence of men smoking, such as South East Asia and the Western Pacific, have a disproportionately low number of articles on masculinity and smoking. Even in those regions covered by articles in this review, there is little exploration of masculinity as a protective factor (especially among children and adolescents). Given the cultural and temporal dynamics of constructed masculinities, there is a need for studies in these areas as a means of incorporating culturally appropriate masculinities in settings-based approaches to smoking-related health promotion programmes, as a catalyst for reducing smoking prevalence among men in such regions.

## Paper context

Despite its high prevalence, smoking among men is not a priority when developing studies and intervention. This review attempts to synthesise current findings for studies on smoking and masculinities, providing a thorough understanding of the possible implementation of masculinity into smoking studies. This study will provide a new perspective on smoking problems, to complement current smoking policies.
